# EBR-YOLO: A Lightweight Detection Method for Non-Motorized Vehicles Based on Drone Aerial Images

**DOI:** 10.3390/s25010196

**Published:** 2025-01-01

**Authors:** Meijia Zhou, Xuefen Wan, Yi Yang, Jie Zhang, Siwen Li, Shubo Zhou, Xueqin Jiang

**Affiliations:** 1 College of Information Science and Technology, Donghua University, Shanghai 201620, China; 2232264@mail.dhu.edu.cn (M.Z.); jz18115112@163.com (J.Z.); 13155830372@163.com (S.L.); xqjiang@dhu.edu.cn (X.J.); 2 College of Computer Science, North China Institute of Science and Technology, Langfang 065201, China; calmerd@ncist.edu.cn

**Keywords:** YOLOv8, lightweight, non-motorized vehicle detection

## Abstract

Modern city construction focuses on developing smart transportation, but the recognition of the large number of non-motorized vehicles in the city is still not sufficient. Compared to fixed recognition equipment, drones have advantages in image acquisition due to their flexibility and maneuverability. With the dataset collected from aerial images taken by drones, this study proposed a novel lightweight architecture for small objection detection based on YOLO framework, named EBR-YOLO. Firstly, since the targets in the application scenario are generally small, the number of Backbone layers is reduced, and the AZML module is proposed to enrich the detail information and enhance the model learning capability. Secondly, the C2f module is reconstructed using part of the convolutional PConv to reduce the network’s computational volume and improve the detection speed. Finally, the downsampling operation is reshaped by combining with the introduced ADown module to further reduce the computational amount of the model. The experimental results show that the algorithm achieves an mAP of 98.9% and an FPS of 89.8 on the self-built dataset of this paper, which is only 0.2% and 0.3 lower compared to the original YOLOv8 network, respectively, and the number of parameters is 70% lower compared to the baseline, which ensures the accuracy and computational speed of the model while reducing its computational volume greatly. At the same time, the model generalization experiments are carried out on the UCAS-AOD and CARPK datasets, and the performance of the model is almost the same as the baseline.

## 1. Introduction

Accompanied by the arrival of the Internet era and the prosperous development of shared travel, non-motorized vehicles, as a kind of efficient and environmentally friendly means of travel, occupy an important component in the modern transport network. According to the China Bicycle Association, the social ownership of electric bicycles in China has reached nearly 300 million, with an annual sales volume of more than 30 million, and the large number of non-motorized vehicles may cause a safety hazard [1]. However, the identification of non-motorized vehicles has rarely been addressed. The traditional ways to monitor non-motorized vehicles include fixed monitoring equipment. This can work the whole day to automatically detect and record illegal acts, but is installed in fixed locations, which cannot cover specific angles or spaces, resulting in some of the illegal acts not being detected. Another traditional way is to send law enforcement officers with mobile enforcement equipment to collect evidence on the spot, who can flexibly respond to various complex traffic environments, but require a large number of human resources. Considering that UAVs have the advantages of wide field of vision, changeable viewpoints, and fast flight speed, they can flexibly cruise over traffic roads, obtain monitoring images from different viewpoints through the adjustment of flight attitude, and cover the blind spots of the fixed monitoring equipment. Also, UAVs can be quickly sent to the areas that need to be monitored, and they can respond quickly to emergencies or special needs. Therefore, the use of UAVs to detect non-motorized vehicles on the road is a preprogram that is worth studying.

Since the basic theory of deep learning was put forward in the last century, many studies have provided different algorithms and models for the recognition rate and accuracy. Currently, the main algorithms in the field of target detection include one-stage algorithms such as SSD [2], YOLO [3], RetinaNet [4], and two-stage algorithms including RCNN [5], FasterRCNN [6], and Mask-RCNN [7]. Two-stage algorithms divide the target into two different stages: first into candidate frame regions, and then the classification and regression of these regions, usually using region-based extraction. Two-stage algorithms have high accuracy and strong detection capability, but have high computational complexity due to slow processing speed and the need to generate a large number of candidate regions. One-stage algorithms usually use an anchor point based detection method based on anchor points. Their advantages include faster processing speeds, excellent real-time performance, and a simplified detection process. In case of road traffic management, real-time capability is crucial as abnormal behavior in road traffic can lead to serious traffic accidents. Given that one-stage algorithms typically exhibit faster inference times than two-stage algorithms, the YOLO family of one-stage algorithms is used as the baseline in this study. However, in the field of real-time detection from a UAV perspective, target detection models are typically constrained by the computational power of the detection terminals, a constraint that poses a significant challenge for model deployment. Optimizing models with a wide range of parameters to deploy on terminals where computational power is a priority remains a key issue in the research of real-time detection from the perspective of drones.

In the field of target detection based on aerial images from drones, researchers have performed many studies. Mohammad Hossein Hamzenejadi and Hadis Mohseni [8] used YOLOv5 as the baseline, reduced the model complexity by using Ghostconv [9] in the Neck, and increased the model accuracy by adding a new detection head and SE attention [10] mechanism, and the resulting model still had a high accuracy at a faster inference speed. Shihai Cao, Ting Wang, Tao Li, and Zehui Mao [11], on the other hand, focused on small target detection; they improved the detection layer and added a multi-scale feature fusion module to improve the accuracy of small targets, and the improved model obtained a large improvement in accuracy. Liqi Yan, Qifan Wang, and Junhan Zhao et al. proposed FPV-NeRF [12], which combined multi-scale keyframe selection and cross-resolution attention mechanisms to achieve the first efficient and detailed 3D scene rendering and prediction in UAV scenes, adapted to the dynamically changing needs of UAV perspectives. Zhang, Jiaqing and Lei, Jie and Xie, Weiying et al. [13] proposed an RSI target detection method, SuperYOLO, which fused multimodal data and used assisted super-resolution learning for high-resolution detection of multi-scale targets. Kim, Munhyeong, Jeong, Jongmin et al. [14] proposed an efficient channel attention pyramid that used transposed convolution for upsampling to avoid greater information loss; however, the method had difficulty extracting multi-scale features in complex backgrounds and performed poorly on small target detection tasks. Zhao, Zheda, Qin, Yong and Qian, Yu et al. integrated the CSP-based Backbone network and detection branch, as well as designing a new loss function to accurately perform the pixel-level parsing task and optimize the coordinate regression process for predicting the bounding box [15]. Wang, Gang and Chen, Yanfei et al. [16] took YOLOv8 as the baseline, and designed a feature processing module FFNB to completely fuse the shallow information with the deep features, and added two new detection heads on this basis, which improved the detection performance of small targets, but this also caused the problem of lower detection rates. Fu Jinyi and Zhang Zijia et al. [17] proposed an improved YOLOv8 algorithm, which enhanced the ability of multi-scale detailed feature acquisition by designing a channel feature partial convolution module, while replacing the detection head and embedding a module that enhanced the ability of context aggregation, which drastically reduced the parameters of the model while improving the detection accuracy by a small margin. To enhance the quality of dateset, Zhichao Chen, Jie Yang and Zhicheng Feng et al. used ChatGPT and AIGC techniques to generate a large number of anomalous images and synthesized the anomalies with normal images by image enhancement methods to address the problem of scarcity of anomalous data [18].

Although the above methods have optimized the performance and complexity of the model to different degrees, further research is needed to deploy the model on edge equipment. In order to make the model meet the demand of lightweight while maintaining a certain degree of accuracy, this paper proposes a EBR-YOLO algorithm to cope with the demand for real-time detection of non-motorized vehicles from the perspective of drones. The main contributions of this algorithm are as follows:

1. Among the three feature layers in the YOLOv8 network structure, P5 is used to detect large targets; however, most of the targets in this paper’s self-constructed dataset are small targets with a pixel area of less than 1024, so this paper considers removing the P5 layer and its associated detection header. Meanwhile, in order to be able to better adapt to the features of this paper’s dataset, this paper improves the feature fusion method in the Neck part, combines the semantic and detailed features of the three layers, and adds the attention module to optimize the performance of the model.
2. Inspired by the FasterNET [19], we design a new structure to reconstruct the C2f module, which performs convolutional computation on only some feature layers, so as to reduce the network’s computational volume and improve the detection speed.
3. We replace the downsample module of the Backbone and Neck part of the original model with the ADown module proposed by YOLOv9 [20], which enhances the ability of the network to retain the target features, and ensures the detection accuracy while improving the detection speed.
4. Compared with some mainstream YOLO series models, as well as other classical detection models, the experimental results demonstrate the superiority of our method. The results on two public datasets also confirm the strong generalization ability of the model. In addition, we perform visual analysis to explain that our proposed method can lighten the model while keeping its efficiency.

## 2. Materials and Methods

### 2.1. Materials

#### 2.1.1. Video Processing

The self-built dataset used in this article is collected from roads in Songjiang District, Shanghai, China. The drone we used was DJI Mini 2, sourced from DJI Technology Co., Ltd., Shenzhen, China, from a height of about 25 m, which can make up for the lack of fixed monitoring equipment. In order to make this dataset more widely applicable, the types of non-motorized vehicles taken in this self-built dataset include bicycles, shared bicycles, and electric bicycles. For video recording, a 3-axis gimbal camera is carried by the drone, with a resolution of 4000×3000. Videos lasting a total of about 2.5 h was obtained. When capturing the videos, the operator will fly the UAV to a designated location and hover in a suitable position for about 15 min each time, and then the operator will control the UAV to fly to the next location to capture the image.

#### 2.1.2. Base Model: YOLOv8

YOLOv8 [21], as a recent research achievement in the field of target detection, has attracted wide attention in the field of deep learning due to its excellent and efficient real-time performance. The algorithm achieves accurate recognition and localization of target objects in images through the optimization of deep neural network architecture and the introduction of a multi-scale prediction mechanism. Its high efficiency is not only reflected in the high speed frame rate, but also in its ability to achieve real-time detection of multiple targets in complex scenes while maintaining high accuracy. Its adaptive anchor frame technique, optimized feature extraction network, and Anchor-Free detection strategy make the algorithm a remarkable breakthrough in the field of target detection.

**Figure 1:**
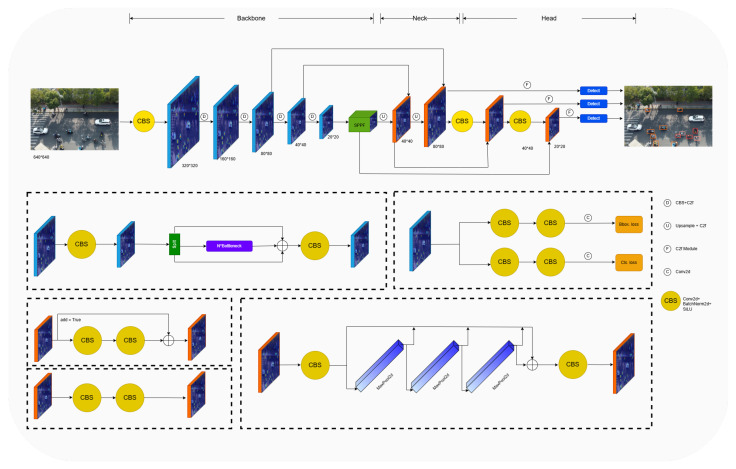
The network structure of YOLOv8.

The structure of the YOLOv8 network model is shown in Figure 1. This design consists of three key components: Backbone, Neck and Head.

Backbone, as the foundation of YOLOv8, undertakes the important task of feature extraction. The two consecutive 3 × 3 convolutional layers allow the network to capture detailed information in the image at different scales. Also, the C2f module enriches the gradient flow of the model, and helps the network to learn features better during the training process. In addition, the SPPF module in Backbone further improves the performance of the network by changing the simple parallel max pooling to serial and parallel.

The Neck part adopts slim-neck [22] design, which optimizes the feature fusion effect through modules such as GSConv, GS bottleneck, etc. GSConv can keep the hidden connections between channels as much as possible, while keeping the time complexity low, so as to enhance the feature representation ability. GS bottleneck, on the other hand, reduces computation while maintaining feature richness by first reducing and then increasing the dimensionality.

Head is the output part of YOLOv8, which is responsible for the final target detection and classification task. In YOLOv8, for the predicted box bounding regression task, distribution focal loss (DFL) [23] and CIoU [24] are employed. Meanwhile, the Anchor-Free detection strategy directly predicts the centroid and size of the target without the preset anchor frame. Also, YOLOv8 uses Global Average Pooling (GAP) [25] for classification. These design makes the detection more concise and efficient while increasing the detection speed.

### 2.2. Methods

**Figure 2:**
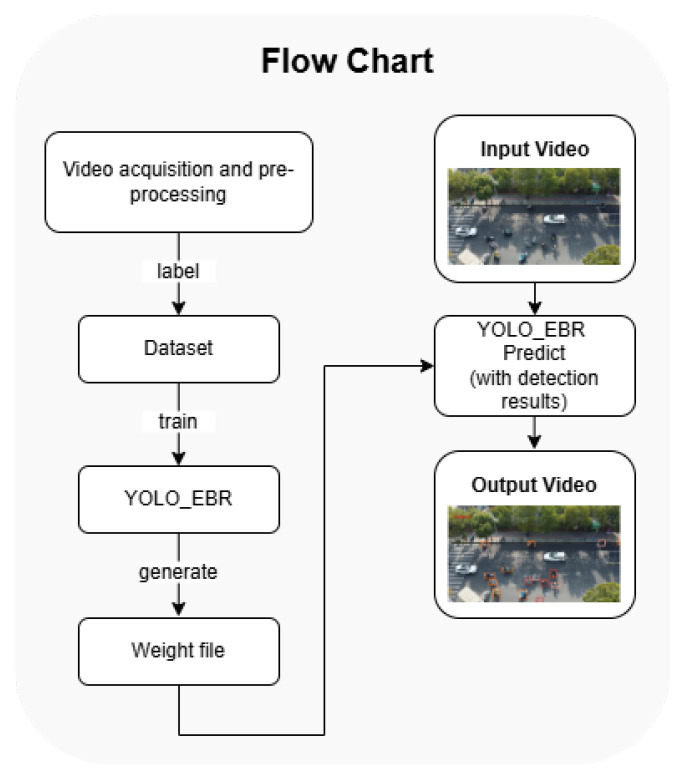
Flowchart of the work including the dataset acquisition, model training, and display of the detection result.

Figure 2 shows the workflow of the method in this paper. To make the images in the self-constructed dataset more relevant to the real situation, we use drones for road image acquisition, including video recording at different locations and different time periods with some other pre-preparations. Then, we manually annotate the targets in the dataset to ensure the quality of the dataset. After that, we train the model and obtain the optimal weights file. Finally, we can use the model and weight files obtained in the above steps to perform target detection on the images, so the input image or video can be processed to output an image or video with a prediction frame.

#### 2.2.1. Image Processing

**Figure 3:**
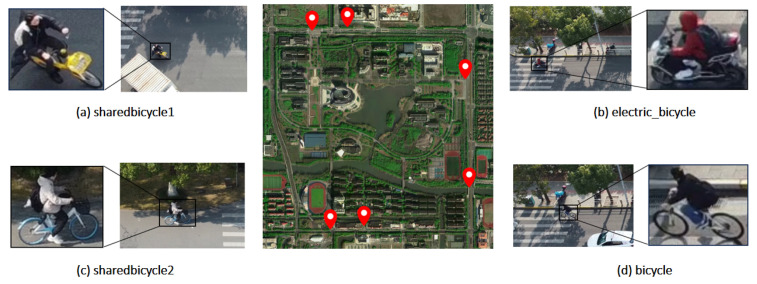
The self-built dataset used in this article, (**a**,**c**) is the two types of shared bicycle owned by companies. (**b**) is the electric bicycle. (**d**) is bicycle owned by people themselves.

To construct the image dataset, this article employed video segmentation, capturing an image every 20 frames and subsequently making selections. A total of 4295 real road images are generated. The LabelImg v1.8.6 tool was used for labeling and, subsequently, files in xml format were generated. In order to enrich the diversity of images in the dataset and adapt to practical application scenarios, the time period for the images includes midday and afternoon, and the weather includes sunny and cloudy days. As a result, the dataset is generalizable, and can be used for non-motorized vehicle identification tasks in different environments, as shown in Figure 3.

Meanwhile, the non-motorized vehicles driving on the road mainly includes bicycles and electric bicycles, and to ensure the model can be widely used, this study further divides the bicycle into shared_bicycle and bicycle, because shared bicycles are under direct management of the enterprise. Therefore, the detection of shared bicycles can help to make certain suggestions for the management decisions of enterprises. The image data were randomly divided into a training set and validation set in a ratio of 8:2, resulting in 3436 training images and 859 validation images.

#### 2.2.2. Improved Model: EBR-YOLO

**Figure 4:**
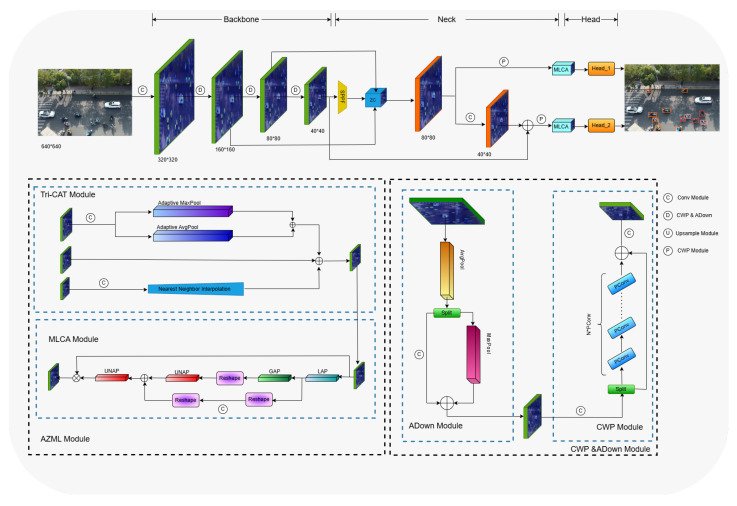
The network structure of EBR-YOLO.

The network structure of EBR-YOLO is shown in Figure 4.

Building upon the YOLOv8 model, this paper introduces the following modifications to create the EBR-YOLO model:

1. Among the three feature layers in the YOLOv8 network structure, P5 is used to detect large targets; however, most of the targets in this paper’s self-constructed dataset are small targets, so this paper considers removing the P5 layer and its associated detection header, by which the parameters can be drastically reduced. Meanwhile, to better adapt to the features of the self-built dataset, this paper proposes a AZML structure to effectively combines the semantic and detail information of three levels features, and improves the model’s learning ability.
2. The PConv module was used as a lightweight improvement to the C2f module in Backbone, which retains the expanded feature extraction capabilities of C2f and replaces the original network structure with a partial bottleneck resulting in a reduction in model parameters and computational complexity.
3. Instead of the downsample module in YOLOv8, the ADown module is used, which consists of multiple convolution and pooling operations to reduce the size of the feature map and increase the number of channels, by which the size of the model can be reduced while retaining as much image information as possible in order for the model to be able to perform target detection more accurately.

#### 2.2.3. Model Structure Optimization

**Figure 5:**
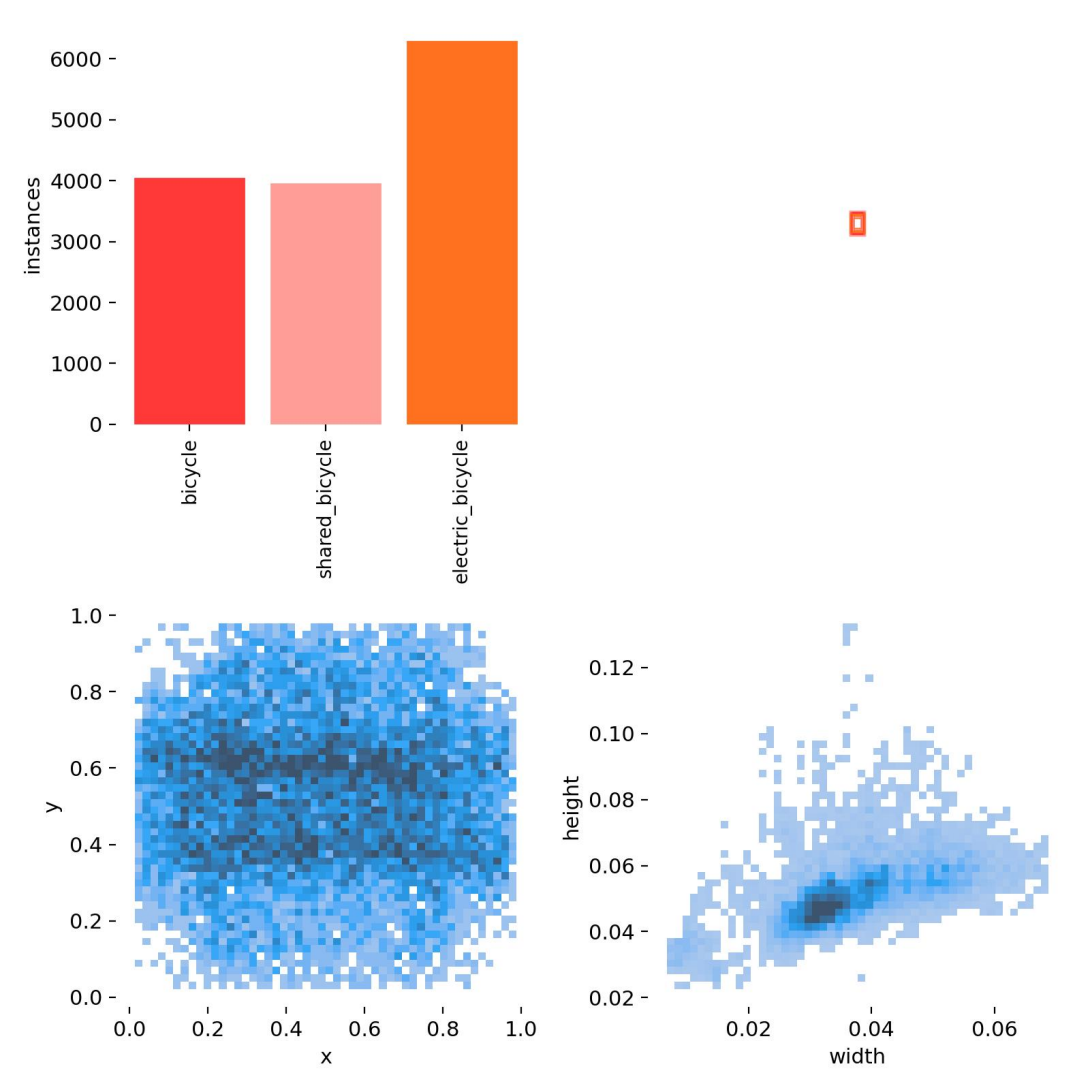
The target pixel area in the self-built dataset.

YOLOv8 will resize the image of the input model to 640×640×3; after 8×, 16×, or 32× downsamplings of its Neck network, the final size of the three feature maps detected are 80×80×128, 40×40×256, and 20×20×512, respectively, due to the fact that most of the targets in this paper’s self-constructed dataset are small targets [26] and the target pixel area is smaller than 0.06 (Figure 5). When the feature map size is compressed to 20×20×512, it will cause a large loss of feature information of the small target, and deep convolution will lead to the information of the small target to be submerged, which will affect the detection accuracy. Therefore, in this paper, the P5 level of the Backbone is removed so that the network model does not undergo too deep convolution. That operation can ensure certain performance requirements for the detection task without bringing complex algorithmic overheads on the self-constructed dataset, and there is a good balance between model complexity and accuracy.

#### 2.2.4. Feature Level Improvement

After cropping the network convolutional layers, we notice that this operation will have some impact on the detection performance of the model. Considering that the detection task in this paper mainly focuses on small targets in the view of UAVs, the feature fusion part needs to be improved to adapt to the dataset. To focus on small target, the high resolution feature layer of the Backbone network is simply added into the Neck network [27,28,29,30]. Although the detection accuracy is improved, the additional down-sampling operation and up-sampling operation will lead to the information loss of small target feature. Since different feature layers of the Backbone network have different sizes, the conventional FPN [31] fusion mechanism only up-samples the small-sized feature maps, and then splits or adds them to the previous layer of features, ignoring the rich information of the larger-sized features; therefore, this paper refers to ASF-YOLO [32] and proposes the Tri-CAT module, which splits the large, medium, and small features, and at the same time, the P2 layer is added to feature fusion operation to better extract the detailed feature information.

**Figure 6:**
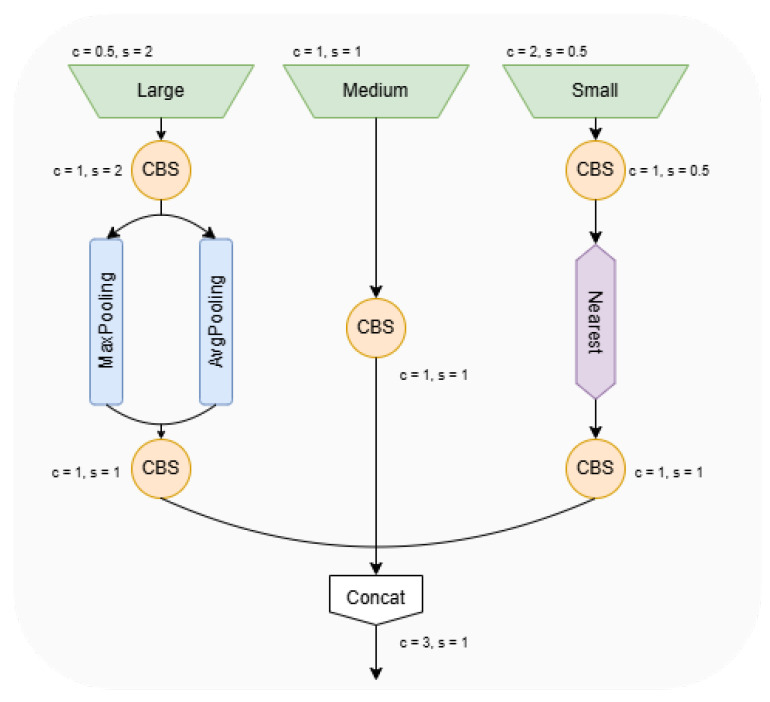
The structure of Tri-CAT module. c represents the number of channels, and s represents the feature map size. The CBS module represents Conv+BN+SiLU operation.

In the large-size feature map part, the number of channels is firstly adjusted using the single-kernel convolution module, and then downsampling is performed using adaptive maximum pooling and average pooling, which helps to retain the effectiveness of high-resolution feature sums, while in the small-size feature map part, the number of channels is also firstly adjusted using the single-kernel convolution, and then upsampling is performed using the closest-neighbor interpolation method. This approach can largely prevent the loss of small target feature information and maintain the richness of local features in low resolution images. The structure of the module is shown in Figure 6.

In order to further enhance the model’s ability to emphasize important elements and suppress irrelevant elements, this paper introduces an attention module in the Neck part, considering that most channel attention mechanisms only contain channel feature information and ignore spatial feature information, while spatial attention modules are usually complex and computationally expensive, which does not meet the purpose of this paper’s lightweight model. In order to strike a balance between performance and complexity, this paper introduces a lightweight hybrid local channel attention module, MLCA [33], to enhance the detection performance of the model, which improves the accuracy by combining channel and spatial attention at both local and global levels while ensuring computational efficiency.

**Figure 7:**
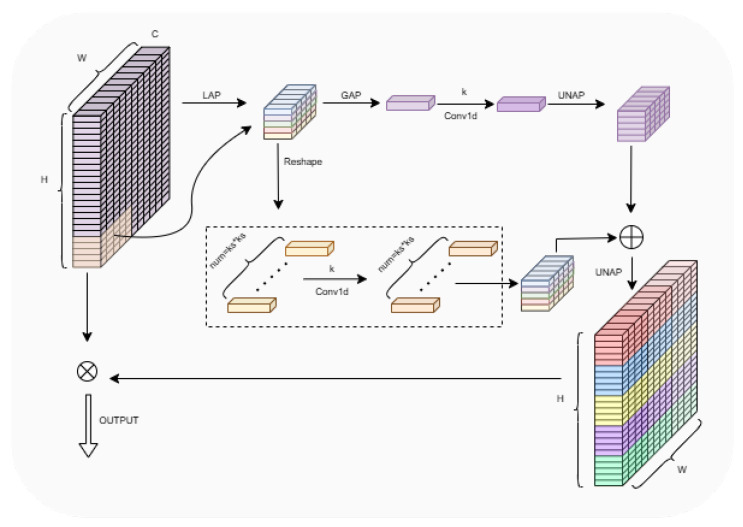
The structure of MLCA.

The module first passes the input feature map through local average pooling (LAP) and global average pooling (GAP) to collect feature information from the local and global regions of the feature map, respectively, and then uses single-kernel convolution and reshape module to perform feature channel compression and rearrangement for subsequent operations. The locally pooled features are combined with the original input features by multiplication operations to strengthen the attention on useful features, while the globally pooled features are combined with the locally pooled features by addition, a step that incorporates the global contextual information into the feature map. Finally, the feature maps after local and global attention processing are again restored to their original spatial dimensions by an inverse pooling operation. The schematic structure of the module is shown in Figure 7.

**Figure 8:**
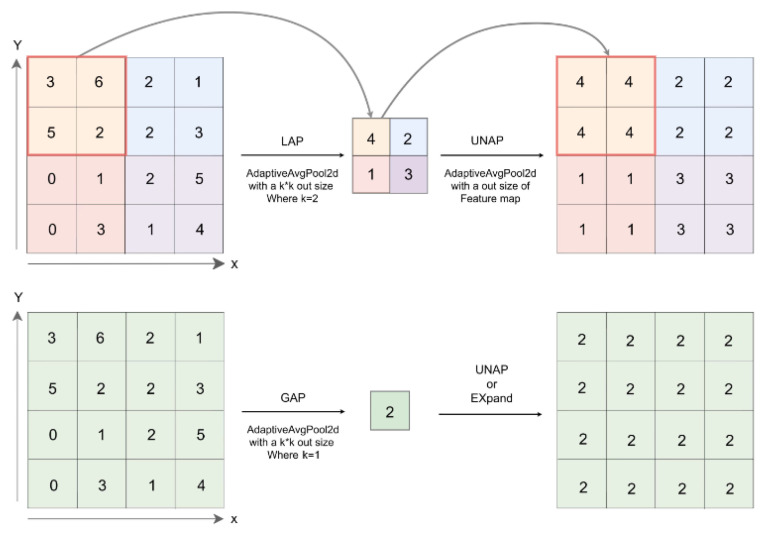
The schematic diagram of GAP, LAP, and UNAP.

Figure 8 depicts the primary characteristics of GAP, LAP, and UNAP. The LAP operation splits the entire feature map into n × k × k patches, and performs average pooling on each of them, which means averaging the values in it. When you need to multiply or add source input directly, you can use UNAP as a kind of anti-average pooling method that focuses on the features of an image and helps to modify it to the needed size. The values in the patch were populated with the weight assigned to the patch. GAP is a global average pooling that uses adaptive average pooling to change the output feature map size to 1 × 1. So unlike LAP, the GAP feature map can be restored to its original size using Expand or UNAP.

#### 2.2.5. Design of Partial Bottleneck

**Figure 9:**
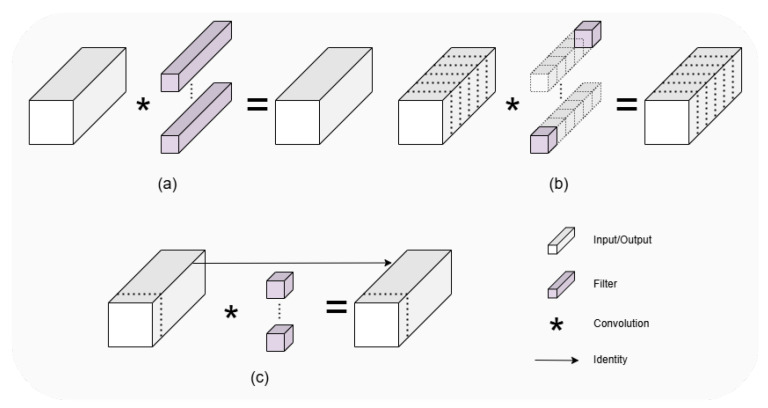
The comparison between common convolution (**a**), depth/group convolution (**b**), and partial convolution (**c**).

The YOLOv8 Backbone network mainly uses C2f module to perform high quality feature extraction from images. Considering that there is a lot of research focusing on the improvement of the network structure, such as the depth separable convolution DWConv [34] for MobileNet [35] and the group convolution Gconv for ShuffleNet [36], these methods can reduce the computation amount and decrease the model performance at the same time. For example, the DWConv module performs independent convolution calculations for each channel of the input layer, and is unable to utilize the feature information of different channels at the same spatial location. FasterNet proposes a new convolutional approach, PConv, to efficiently extract features by reducing computation and memory accesses. The schematic diagram is shown in Figure 9. PConv uses regular convolution for feature extraction for only some of the input channels, leaving the rest of the channels unchanged since, for contiguous or regular memory access, the first or last consecutive cp channels are considered as the representatives of the whole feature maps for computation. Though there are only cp channels used for spatial feature extraction, the remaining channels are untouched, instead of being removed. AS a result, PConv has less floating-point computation than regular convolution, and can extract spatial features efficiently.

**Figure 10:**
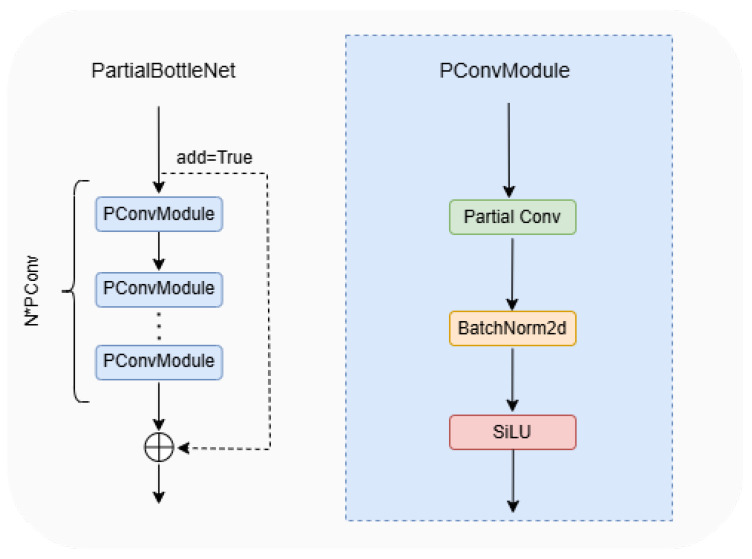
The structure of the partial bottleneck (**left**) and PConv modules (**right**).

The basic convolutional module of PCTF module uses PConv to lighten and improve the C2f module, which retains the extended feature extraction function of C2f and uses part of the convolutional bottleneck to replace the original network structure. The partial convolution bottleneck structure is shown in Figure 10. The bottleneck structure consists of two PConvModel modules, the first one compresses the features, and the second one is responsible for feature high-dimensional mapping and fusion with the input.

#### 2.2.6. ADown

**Figure 11:**
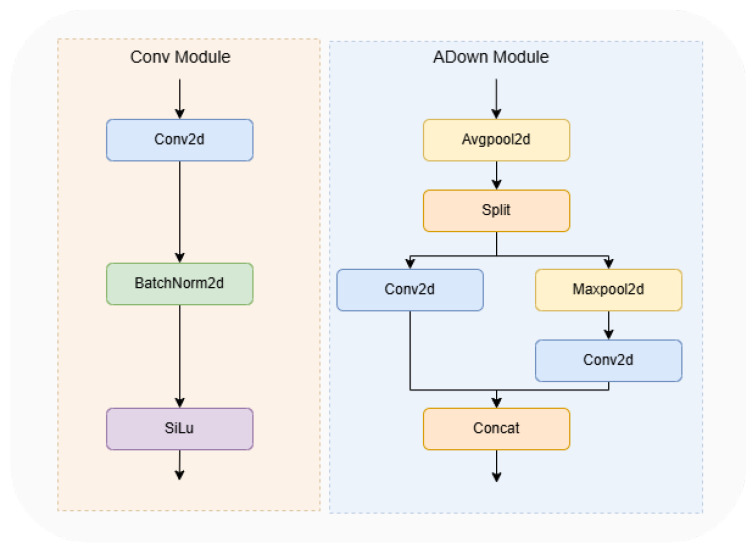
A comparison of the Conv and ADown modules.

This article uses the ADown module instead of the normal convolutional module in YOLOv8. The ADown module is a fast convolution for down-sampling operations in the target detection task. Downsampling is equivalent to a pooling operation on the feature maps, which can make the later convolutional kernel learn more global information. In deep learning, the sensory field refers to the amount of convolutional neural networks that each output feature point is able to see in the the size of the region of the input image. A larger receptive field helps the model capture a larger range of contextual information in the image, which improves the accuracy of target detection. Meanwhile, the ADown module prevents overfitting to a certain extent by reducing the size of the feature map and the number of parameters. The ADown module separates the inputs by average pooling, and then performs the convolution and pooling operations on the two channels separately, and finally merges them together. The ADown structure is illustrated in Figure 11. Although the ADown module drastically reduces the model complexity by reducing the number of parameters, its design also focuses on retaining as much image information as possible, so that the model can perform target detection more accurately.

## 3. Results

### 3.1. Experimental Settings

The hardware platform and environmental parameters used in the experiment are shown in Table 1.

To facilitate flexible deployment on hardware devices in various application scenarios, the YOLOv8 model has been adapted to generate five different scaled models by adjusting two parameters: width and depth. These models are referred to as YOLOv8n, YOLOv8s, YOLOv8m, YOLOv8l, and YOLOv8x. The parameters and resource consumption of the five models increase sequentially, and the detection performance becomes better and better. The width, depth, and maximum number of channels corresponding to these five models are shown in Table 2.

The experimental training parameter settings are as follows: a batch size of 2 is selected, the input image resolution is set at 640 × 640, and the number of epochs is specified as 300. The learning rate is set to 0.01. Additionally, the depth scale parameter is assigned a value of 0.33, while the width scale parameter is set to 0.25. The training parameters set are shown in Table 3.

### 3.2. Evaluation Indices

To select the optimal model, this article employed various metrics to access its performance, including parameters, GFLOPs, time, Precision(P), Recall(R), mean Average Precision(mAP) and model size. The specific meanings of some of the above indicators are as follows:

1. Precision rate: Precision represents the proportion of predicted true positive cases to all predicted positive cases. It is used to measure the probability of a positive example being correctly predicted in the prediction results.
  $$Precision=\frac{TP}{TP+FP}$$
2. Recall rate: Recall rate represents the proportion of predicted true positive samples to the total number of actual positive samples, used to reflect missed recognitions.
  $$Recall=\frac{TP}{TP+FN}$$
3. Average precision value: This is calculated as the area under the Precision–Recall curve in a coordinate graph with precision on the vertical axis and recall on the horizontal axis. The mean average precision (mAP) is the average AP across all categories in the dataset, calculated using the formula below. The threshold for general object recognition is set at 50%. This means that a prediction box with an IoU greater than 0.5 is considered valid. The term mAP@50:95 refers to the IoU values ranging from 50% to 95%, with mAP values calculated every 0.05. The mean of all mAPs calculated is then taken.
  $$mAP=\frac{1}{N}\sum_{i=1}^{N}AP_{i}$$

### 3.3. Cross Validation

To assess the model’s performance and stability, and to ensure the randomness of our dataset while minimizing biases from data splitting, we employ a *k*-folds cross-validation approach [37] in this section. Given our dataset’s 8:2 division, we opt for a 5-fold cross-validation strategy. The dataset is randomly partitioned into five segments, with each segment serving as the validation set once while the remaining segments constitute the training set. This method allows us to test the model’s robustness. During the evaluation, we utilize the bias–variance trade-off to address potential issues. Given *k* validation folds within $D_{train}$, we can calculate *k* performance scores, *v*, and average them. In a validation fold *i*, we call one performance score $v_{i}$,

$${\bar{v}}_{train}=\frac{1}{k}\sum_{i=1}^{k}v_{i}$$

To indicate the robustness of the model during training, we can also calculate the standard deviation as a function of *v*,

$$\sigma \left(v_{train}\right)=\sqrt{\frac{1}{k}\sum_{i=1}^{k}{\left(v_{i}-{\bar{v}}_{train}\right)}^{2}}$$

**Figure 12:**
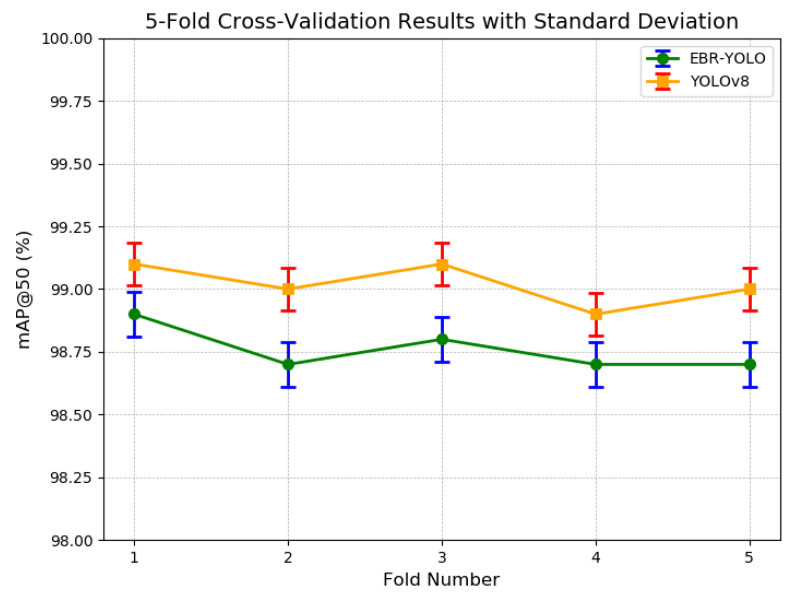
Visualization of cross validation.

Finally, we can determine the model performance through the average performance and the standard deviation of the model between folds during training. From the result in Figure 12 we can find that the model has a small range of fluctuation and good stability on our self-built dataset.

### 3.4. Ablation Experiments

Table 4 shows the results of the ablation experiments on the self-constructed dataset. From the results, each improvement enhances the detection performance of the network. The operation of cropping the P5 feature layer to the network shows that the improvement in this paper makes the algorithmic complexity of the model reduced to a great extent, but the detection accuracy does not change much. The introduction of the AZML feature fusion operation allows the model to enhance its ability to recognize small targets, obtaining a better model performance at the cost of a small number of parameter additions. The bottleneck structure incorporated into PConv is able to further reduce the number of network parameters by a significant amount of 361,248 while, at the same time, reducing the computation amount of 3.8 GFLOPs, which means that using the improved structure of PConv module does not affect the feature extraction effect of the network, and can balance the computation and detection effect of the network. The ADown module, which is used to replace the ordinary downsample operation, not only has an effect on lightweight model, but at the same time, its enhancement and retention of the feature operations also helps the network’s effect on the detection of small targets. It makes the network improve a little under the index of mAP@50.

The improved network reduces to 943,702 and 6.7 in Parameter and GFLOPs. Meanwhile, it only reduces 0.2% and 0.4% in its mAP@50 and mAP@50:95 metrics, respectively. The experimental results show that the design scheme of EBR-YOLO can reduce the number of parameters and computation by reducing redundant computations and memory accesses, and it can maintain the effect on feature extraction, and the detection accuracy on self-constructed dataset is almost the same as that of the baseline.

### 3.5. Comparative Experiments

To better evaluate EBR-YOLO, the lightweight detection method for non-motorized vehicles from the perspective of drones proposed in this paper, the selected performance metrics of the proposed method are compared with two-stage object detection method, including FasterRCNN [38] and the state-of-the-art CNN-based one-stage object detection methods, including YOLOv3-tiny [39], YOLOv5 [40], YOLOv11n [41], SSD, and RT-DETR [42] on the self-constructed dataset.

The recognition results of the algorithms mentioned above are shown in Table 5. In terms of mAP@50, the proposed method achieved 98.9%, which is 14.2%, 13.5%, and 20.6% better than FasterRCNN (Resnet50), FasterRCNN (VGG), and SSD (VGG), respectively. In terms of parameters and GFLOPs, the proposed method performs the best. From the result, it can be also seen that, among all of the models, YOLOv8’s model has the least number of parameters and GFLOPs, while its average accuracy can be ranked second only to YOLOv5s, while the average accuracy of the model proposed in this paper can be ranked third, the same as that of YOLOv3_tiny, but its number of parameters is only about 70% of the YOLOv8 model. Overall, although YOLOv5s can achieve the best detection accuracy, and YOLOv8n, YOLOv11n, RT-DETR, and YOLOv3_tiny are also excellent, their number of parameters are too large, and they are not able to satisfy the task of real-time detection on the edge side, while the Faster R-CNN and SSD series of algorithms are not able to satisfy the demand in terms of both accuracy and size. Comparing the parameters, GFLOPs, and mAP@50, the EBR-YOLO algorithm has better performance.

## 4. Discussion

### 4.1. Visualization and Analysis of Experimental Results

**Figure 13:**
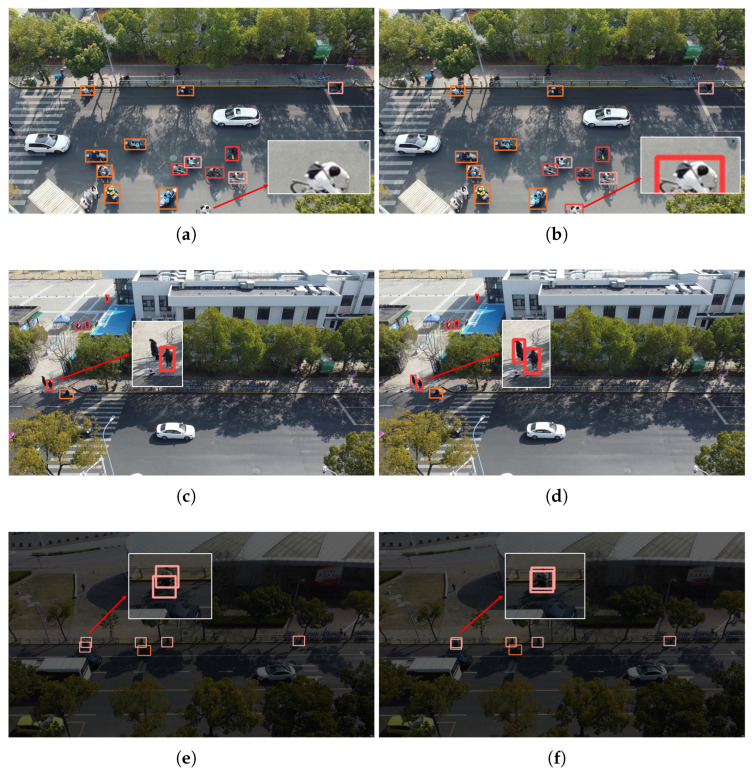

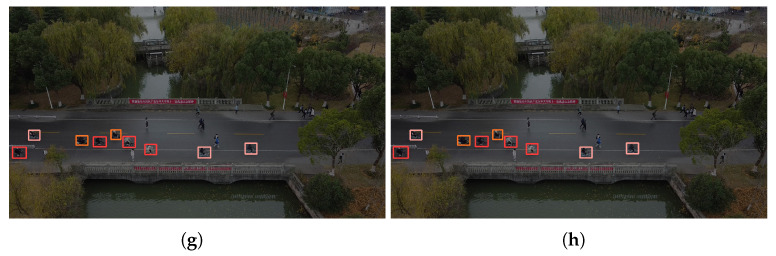
Comparison of detection results in different scenarios. (**a**,**c**,**e**,**g**) The results of YOLOv8 model detections; (**b**,**d**,**f**,**h**) The results of EBR-YOLO model detection.

In order to better demonstrate the detection effect of the improved network, UAV aerial images under three different scenarios are chosen in this paper, and the experimental results are shown in Figure 13. The first row is the detection scene of dense small targets under sufficient light, the second row is the detection scene under complex background, and the third and fourth rows are the detection scene under dim environment. The comparison results show that the detection effect of EBR-YOLO is almost the same as that of YOLOv8 in different scenarios, and, as can be seen in the first row, the improved network shows better detection ability when detecting the targets at the edge of the image, i.e., the cut targets. However, it can be seen in the second line that the feature extraction ability of the improved network is slightly inferior when facing small targets with unclear features, this is due to the fact that the feature information of the wheels is very weak, and the improved model uses the idea of partial convolution to improve the detection speed, and calculates with a certain channel regarded as a representative of the whole feature map, so it is slightly weaker in detecting certain targets with unclear features. Meanwhile, in the third and fourth rows, we can see that in a dim environment, the detection effect of the improved network is the same as that of the baseline. However, in the dim environment, facing the existence of overlapping detection targets, the detection frame of the improved network appears to be slightly shifted; however, it is still able to identify the target object. It is proved that the improved network can maintain good detection effect while increasing the detection speed.

### 4.2. Comparative Experimental Validation of Model Generalizability

In order to verify that the improved algorithm proposed in this paper has a significant detection effect and good generalization on the task of small target recognition in other aerial photography viewpoints, this paper considers conducting the comparative experiments between YOLOv8 and EBR-YOLO proposed in this paper on other public datasets. Considering that the detection task in this paper is characterized by small targets and few classifications, the public datasets with similar characteristics are the UCAS-AOD [43] dataset and CARPK [44] dataset, which are selected for the experiment. The following are the experiments on the two public datasets:

(1) UCAS-AOD: UCAS-AOD collected thousands of high-resolution aerial images containing samples of two classes (aircraft and automobiles), as well as a certain number of counterexample samples, and contains a total of 2420 images and 14,596 instances from various heights. These images also contain complex background environments, all of which have been carefully selected so that the object orientations in the dataset are evenly distributed. From Table 6, it can be seen that, compared to YOLOv8, the improved model proposed in this paper dramatically accelerates the computational speed of the model, and reduces the large number model parameters while sacrificing a very small amount of accuracy, which proves that EBR-YOLO, proposed in this paper, has a good generality.
(2) CARPK: CARPK consists of a dataset of more than 90,000 instance objects collected by a UAVs at an altitude of 40 m. The detection targets of this dataset include vehicles in a car park give, and each vehicle is labeled with a bounding box to mark the image set. Table 6 shows the experimental results; it can be seen that the algorithm proposed in this paper achieves the same detection accuracy as YOLOv8 on this dataset using less parameters and a faster speed, which proves the generalization of the proposed algorithm and the excellent detection results.

**Figure 14:**
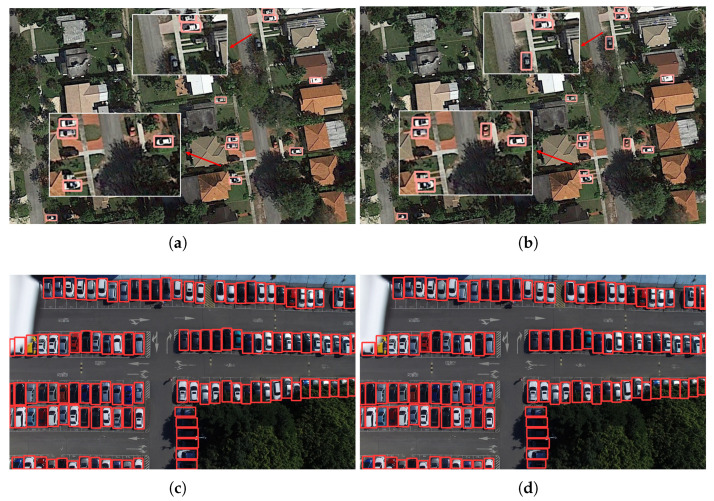
Comparison of detection results in two datasets. (**a**,**c**) The results of YOLOv8 model detections; (**b**,**d**) The results of EBR-YOLO model detection.

The images in the above two datasets are randomly selected for visual effect comparison verification, as shown in Figure 14. The YOLOv8 algorithm is shown on the left, and the EBR-YOLO algorithm is shown on the right, and it can be seen from the first row that the EBR-YOLO algorithm detects targets missed by the YOLOv8n algorithm in complex backgrounds on the UCAS-AOD dataset. From the second row, the detection effect of the two algorithms is almost the same on the CARPK dataset. The result indicates that the EBR-YOLO algorithm achieves almost the same detection effect as baseline with a smaller model size in other aerial viewpoints of the small target recognition task with a strong generalization ability and versatility.

## 5. Conclusions

In this paper, from the perspective of UAV target detection algorithm design, combined with the self-constructed aerial image dataset under the UAV viewpoint, EBR-YOLO is proposed for adaptation to mobile devices of UAV systems. First, considering that the targets in the dataset are small targets, this paper removes the detection layer focusing on large targets, which greatly improves the network computation speed while ensuring the detection accuracy; for the characteristics of the dataset, the AZML structure is designed to reshape the structure of Neck in order to effectively combine the semantic and detail information of the high, medium, and low-level features, and improve the model’s learning ability. By using the characteristics of the PConv structure to further reduce the complexity of the model, we propose using the ADown structure to achieve a larger sensing field and retain as much image information as possible with a small number of computational parameters. The results of the ablation experiments show that the modules have a significant effect on both maintaining the detection accuracy of the model, and reducing the computational complexity of the model. The results of the ablation experiments show that the improvements and modules made are effective in maintaining the model detection accuracy and reducing the computational complexity of the model. The algorithm proposed in this paper can significantly reduce the complexity of the YOLOv8s algorithm, the detection speed can meet the goal of placing the network on mobile devices, and it can also ensure a fairly high detection accuracy. Compared with the current mainstream classical target detection algorithms, it can be seen that the algorithm proposed in this paper achieves a balance between detection accuracy and detection speed, and has certain superiority. The mAP@50 of the final algorithm model reaches 98.9%, and the detection speed of the model reaches 89.4 FPS, which is able to maintain almost the same detection performance while reducing the number of parameters by almost 70% compared to baseline. The algorithm is also validated by generalization experiments on the UCAS-AOD and CARPK datasets with better results. As a result, the small target detection algorithm proposed in this paper can be regarded as a generalized lightweight detection algorithm for fewer categories of small targets with significant results.

## Figures and Tables

**Table 1:** Test platform.

Name	Version
OS	Windows 11
CPU	Intel(R) CPU E3-1230 v3 @ 3.30GHz
GPU	NVIDIA Geforce GTX 1660
Python3	Python 3.8 based on Anaconda
CUDA	11.0.228
YOLO	8.0.48

**Table 2:** Parameters corresponding to different sizes of YOLOv8.

Model	Depth	Width	Max Channels
YOLOv8n	0.33	0.25	1024
YOLOv8s	0.33	0.50	1024
YOLOv8m	0.67	0.75	768
YOLOv8l	1.00	1.00	512
YOLOv8x	1.00	1.25	512

**Table 3:** Training parameters set during model training.

Name	Image Size	Initial Rate	Epoch	Batch Size
Parameter	640 × 640	0.01	300	2

**Table 4:** The results of the ablation experiments. ✓ indicates that we have incorporated this change, while ✕ signifies that we have not applied this change.

Number	Cut	AZML	CSP	ADown	Parameters	GFLOPs	FPS	mAP@50	mAP50:95	Size (MB)
1	✕	✕	✕	✕	3,011,433	8.2	91.4	99.1	82.8	5.96
2	✓	✕	✕	✕	1,101,798	6.7	110.7	98.5	78.6	2.33
3	✓	✓	✕	✕	1,304,950	8.1	95.6	98.7	78.6	1.62
4	✓	✓	✓	✕	1,040,214	7.3	102.5	98.7	79.5	2.01
5	✓	✓	✓	✓	943,702	6.7	89.4	98.9	79.8	2.01

**Table 5:** Comparison of performances of different models on self-built dataset. The content in bold represents the optimal results under that metric.

Name	Parameters	GFLOPs	mAP@50
SSD(VGG)	26,285,486	62.75	78.2
FasterRCNN(Resnet50)	28,295,818	948.14	84.6
FasterRCNN(VGG)	137,098,724	370.21	85.3
YOLOv3_tiny	8,674,496	13.6	98.8
YOLOv5s	7,068,936	16.5	**99.5**
YOLOv8n	3,011,433	8.2	99.1
YOLOv11n	2,582,737	8.1	99.3
RT-DETR	32,812,241	128.2	98.2
EBR-YOLO	**943,702**	**6.7**	98.9

**Table 6:** Comparison of performances of YOLOv8 and EBR-YOLO on UCAS-AOD and CARPK.

Dataset	Model	P/%	R/%	mAP@50/%	Size (MB)
UCAS-AOD	YOLOv8n	95.6	93.11	97.7	6.0
	EBR-YOLO	95.3	92.89	97.3	2.0
CARPK	YOLOv8n	99.1	98.57	99.4	6.0
	EBR-YOLO	98.95	97.75	99.3	2.0

## Data Availability

The original contributions presented in the study are included in the article.
